# Interplay between lipid profile and anthropometric measures as indicators of cardiometabolic risk in women with polycystic ovary syndrome

**DOI:** 10.3389/fendo.2024.1398017

**Published:** 2024-11-20

**Authors:** Marzena Jabczyk, Justyna Nowak, Paweł Jagielski, Bartosz Hudzik, Jakub Borszcz, Barbara Zubelewicz-Szkodzińska

**Affiliations:** ^1^ Department of Nutrition-Related Disease Prevention, Medical University of Silesia, Bytom, Poland; ^2^ Department of Metabolic Disease Prevention, Medical University of Silesia, Bytom, Poland; ^3^ Department of Cardiovascular Disease Prevention, Medical University of Silesia, Bytom, Poland; ^4^ Department of Nutrition and Drug Research, Institute of Public Health, Faculty of Health Science, Jagiellonian University Medical Collage, Karków, Poland; ^5^ Third Department of Cardiology, Silesian Centre for Heart Disease, Faculty of Medical Science in Zabrze, Medical University of Silesia, Zabrze, Poland; ^6^ Student Scientific Circle Affiliated of Department of Metabolic Disease Prevention, Faculty of Public Health in Bytom, Medical University of Silesia, Bytom, Poland; ^7^ Department of Endocrinology, District Hospital, Piekary Śląskie, Poland

**Keywords:** polycystic ovary syndrome, lipid disorders, cardiometabolic risk factors, anthropometry, metabolic profile

## Abstract

**Objectives:**

Polycystic ovary syndrome (PCOS) is a complex endocrine disorder that often coexists with cardiometabolic risk factors. Women with PCOS have a two-fold increased risk of developing type 2 diabetes and substantially elevated risk for cardiovascular disease (CVD) events later in life. PCOS patients may require more comprehensive metabolic screening to identify populations at higher risk of developing CVD and dyslipidemia. It is recommended to evaluate lipid profile, glucose tolerance and of women with PCOS every 2-3 years. Simple, short, and easy methods for the assessment of CVD risk in women with PCOS may be useful tools for implementing CVD prevention strategies by doctors or nutritionists. The aim of this study was to investigate the usefulness of anthropometric indices in the assessment of cardiometabolic risk based on lipid profile in patients with PCOS.

**Material and methods:**

The study involved 49 of Caucasian women aged 18-39 who were diagnosed with PCOS based on the Rotterdam criteria and divided into two groups with normal lipid profile (N=14) and dyslipidemia (N=35). Biochemical parameters were tested in the morning while fasting. Anthropometric parameters such as Body Mass Index (BMI), Body Adiposity Index (BAI), Waist-to-Hip Ratio (WHR), and Waist-to-Height Ratio (WHtR) were calculated, while the Percent of Body Fat was measured using a body analyzer.

**Results:**

The study demonstrated that women with dyslipidemia were older than the control group, 33 years (27-37) *vs* 24 years (21-26), p<0.01. Neither BMI nor BAI (%) correlated with total cholesterol (p=0.63 and p=0.27). Other lipid parameters, such as serum HDL cholesterol (R=-0.68, p<0.01; R=-0.58, p<0.01), LDL cholesterol (R=0.34, p=0.02; R=0.37, p=0.01), non-HDL cholesterol (R=0.40, p<0.01; R=0.42, p<0.01), and triglycerides (R=0.56, p<0.01; R=0.51, p<0.01) correlated with BMI and BAI (%). ROC analysis demonstrated a high predictive value for age in identifying dyslipidemia. ROC analysis demonstrated poor predictive value for BMI, BAI, WHR, WHtR in identifying dyslipidemia.

**Conclusions:**

Analysis of simple and rapid parameters used to assess body fat, such as BMI, BAI, WHR, and WHtR, has shown that they are poor predictors of dyslipidemia in women with PCOS. In young women with PCOS, age appears to be a more reliable predictor of dyslipidemia.

## Introduction

Cardiovascular disease (CVD) is the leading cause of mortality and morbidity worldwide, accounting for more than 31 million of deaths each year (1, 2). There are numerous risk factors of CVD, including male sex, age, excessive body mass (obesity), lipid disorders, hypertension, and diabetes (2). Polycystic ovary syndrome (PCOS) is an endocrine disorder manifested by ovulatory dysfunction, hyperandrogenism, and polycystic-appearing ovaries morphology.

Women with PCOS have a two-fold increased risk of developing type 2 diabetes (T2DM) and substantially elevated risk for CVD events later in life (3, 4). Since women with PCOS are typically within the pre-menopausal population, long-term CVD risk assessment should be given high priority (3, 4).

Excessive body fat is recognized as a metabolic disease (5). The parameter most strongly associated with cardiometabolic risk factors is visceral adipose tissue (6). Visceral adiposity is associated with dyslipidemia, hypertension, insulin resistance and may be a contributing factor in the development of CVD (6, 7). Reproductive abnormalities are more frequently related to adipose tissue and obesity and are present in 7-10% of women of reproductive age (7). A simple index of weight-for-height, known as body mass index (BMI), is commonly used to classify overweight and obesity in general population (8). Obesity may be attributed to insulin resistance (IR) leading to hyperinsulinemia which stimulates ovarian steroidogenesis (10). Chronic exposure to androgens is associated with the accumulation of visceral fat, which leads to central obesity (3, 9–11). Moreover, elevated testosterone levels and increased BMI have a substantial correlation with diabetes in women with PCOS (12). PCOS patients may require more comprehensive metabolic screening to identify populations at higher risk of developing CVD and dyslipidemia (13). It is recommended to evaluate lipid profile, glucose tolerance and of women with PCOS every 2-3 years (13). Simple, short, and easy methods for the assessment of CVD risk in women with PCOS may be useful tools for implementing CVD prevention strategies by doctors or nutritionists (13).

## Objectives

The aim of this study was to investigate the usefulness of anthropometric indices in the assessment of cardiometabolic risk based on lipid profile in patients with PCOS.

## Materials and methods

### Study participants

This cross-sectional study was conducted from 2015 to 2018 in the Department of Endocrinology, Piekary Medical Centre, St. Luke’s Local Hospital in Piekary Śląskie, Poland. Inclusion criteria were: PCOS diagnosis based on the 2004 Rotterdam criteria (14), age between 18–40 years and consent to participate. Exclusion criteria included: presence of a cardiac electronic implantable devices, pregnancy, lower limbs prostheses, feet dressings, the use of hormonal contraceptives, glucocorticosteroids, oral steroid medications, lipid-lowering drugs, or drugs that affect glucose metabolism, previous diagnosis and treatment of diabetes mellitus, decompensated thyroid disorders, androgen excess disorders (congenital or late-onset congenital adrenal hyperplasia, hyperprolactinemia, Cushing’s disease/syndrome, and androgen-secreting tumors, idiopathic hirsutism), depressive disorders and ongoing treatment of depression, and rehospitalization lack of patient consent to participate in the study.

**Figure 1:**
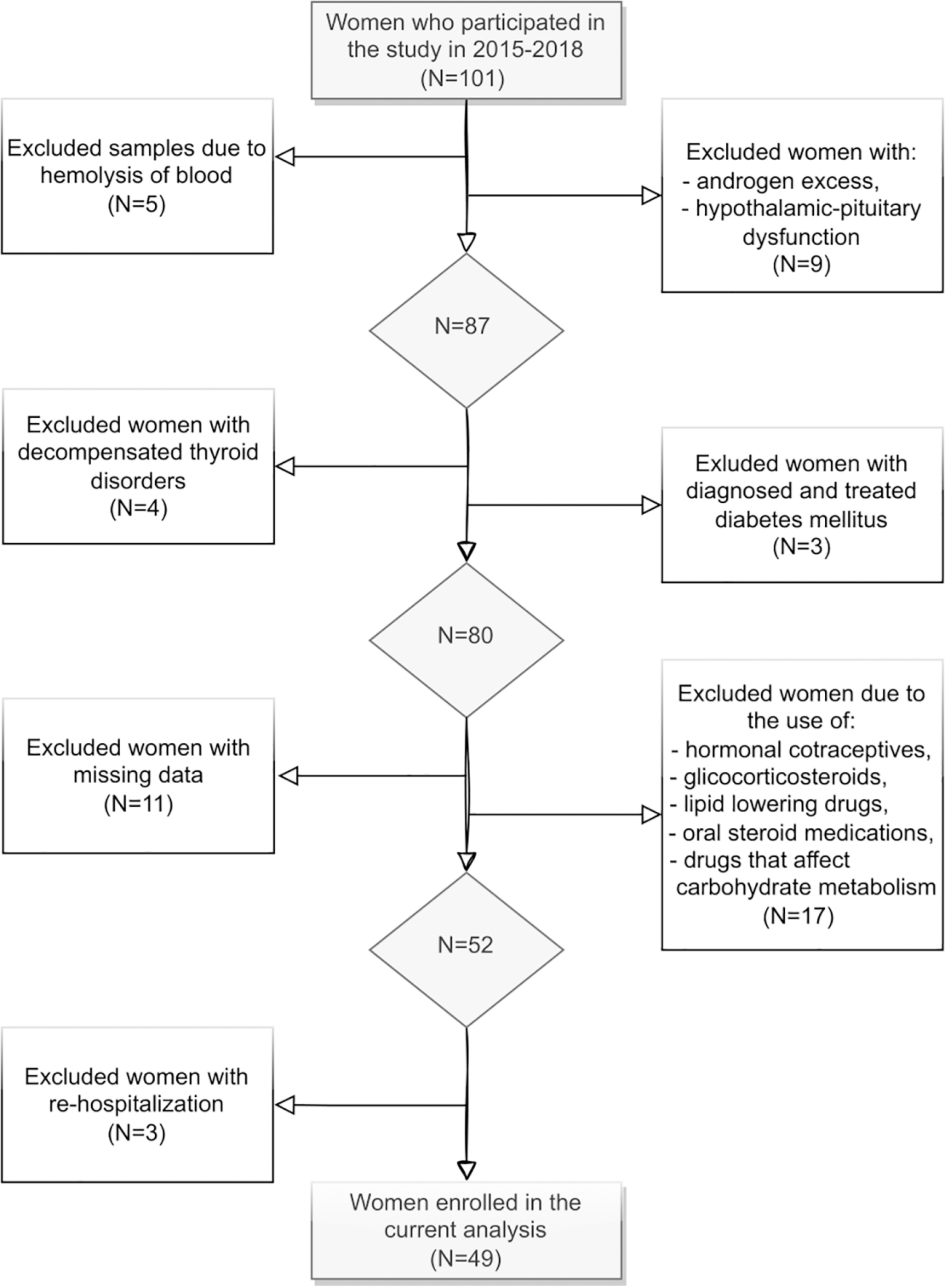
Selection of study participants.

101 women aged 18-39, with a median age of 25 years were screened. The final study sample included 49 women (**Figure 1**). Ultimately, the study population was divided into two groups: those with a normal lipid profile (N=14) and those with dyslipidemia (N=35).

The study follows to the principles outlined in the Declaration of Helsinki and was reviewed and approved by the local bioethics committee (KNW/0022/KB1/143/15). Data analysis consent was obtained from all participants.

### Data collection

Fasting blood samples were collected in the morning. A 1 ml blood sample collected as part of routine tests was preserved for further analysis. After centrifugation, the samples were frozen and stored at -70 degrees Celsius until for further analyses. Laboratory tests used in the study were performed following the Standard Work Instructions (SWI) at KORLAB Medical Laboratories, NZOZ Rudzkie Laboratoria Analityczno-Bakteriologiczne s.c. Testosterone levels were measured using the CMIA method - chemiluminescent microparticle immunoassay on the Abbott Alinity platform. For the determination of glucose concentration, a reference enzymatic method with hexokinase was used, utilizing a Cobas Integra 400+ automated analyzer. The glycated hemoglobin was analyzed using high pressure liquid chromatography HPLC (chromatography reversed-phase cation exchange chromatography). An ADAMS A1c HA-8180 analyser was used for analysis. To determine fasting insulin, the ARCHITECT insulin assay was employed, which is a one-step immunochemical assay for the detection of human insulin in plasma or serum using chemiluminescent microparticle immunoassay (CMIA) and microparticle tracer. To determine total cholesterol concentration a Cobas Integra 400+ quantitative analyzed was used, which used the enzymatic-calorimetric method. The direct quantitative determination of cholesterol LDL-C was achieved using the MULTIGENT Direct LDL assay. This test provides a method for direct measurement of serum or plasma levels of cholesterol LDL, without the need for off-analyser pretreatment or centrifugation. An Architect c8000 analyser was used for the determination. The quantitative determination of cholesterol HDL concentration was accomplished by employing a homogeneous calorimetric enzymatic technique. A Cobas Integra 400+ analyser was used. The Cobas Integra 400+ analyser was employed to determine the concentration of using triglyceride concentration using an enzymatic-calorimetric method utilizing oxidase glycerophosphate and 4-aminophenazone.

Biochemical parameters: glucose levels, insulin levels, glycated hemoglobin A1c (HbA1c), total cholesterol (TC), high-density lipoprotein (HDL), low-density lipoprotein (LDL), triglycerides (TG) were determined and used to calculate the indices. Dyslipidemia was diagnosed based on the following criteria: total serum cholesterol > 5.00 mmol/L and/or LDL-C >3.00 mmol/L and/or serum triglycerides >1.7 mmol/L and/or HDL-C <1.2 mmol/L (15).

The anthropometric indices were calculated according to World Health Organization (WHO) norms for BMI (8) and according to the cut-offs proposed by Bergman for BAI (16).

BMI was calculated according to the following formula:


$$\text{BMI}=body\;weight\;\left[kg\right]/height\;{\left[m\right]}^{2}$$

The following formula was used for the computation of BAI:


$$\text{BAI}=\left(hip\;circumference\;\left[cm\right]/height\;{\left[m\right]}^{1.5}\right)–18$$

Body composition analysis was performed using the TANITA BC 420 MA analyser (TANITA, Japan) with a certificate MDD 93/42 EEC for medical devices.

### PCOS diagnosis

All women in the study met the 2004 Rotterdam criteria for the diagnosis of PCOS (14), which required the presence of at least two of the three following clinical abnormalities: polycystic ovarian morphology on ultrasound exam – 12 or more follicles of 2-9 mm in diameter in each ovary and/or increased ovary volume >10 ml (17), clinical and/or biochemical hyperandrogenism (testosterone >70 ng/dl) (18), amenorrhea or oligomenorrhea. A transvaginal ultrasound was performed using a transducer with a bandwidth frequency range of 6-12 MHz, which is optimal for detailed visualization of ovarian structure in the diagnosis of polycystic ovary morphology.

Hyperandrogenism was diagnosed based on elevated androgens (testosterone and/or androstenedione) levels and/or the presence of clinical features (increased body and facial hair – hair distribution and hair growth intensity of nine different body region in Ferriman-Gallway score of >8) (19). Free androgen Index (FAI) has also been evaluated. Other causes of hyperandrogenism include congenital adrenal hyperplasia, hyperprolactinemia, Cushing’s disease, certain types of cancer and certain medications were excluded (see exclusion criteria).

### Statistical analysis

Statistical analysis was performed using the STATISTICA 13 PL software (Tulsa, Oklahoma, OK, USA). The Shapiro-Wilk test was used to test the distribution. Continuous variables are expressed as means ± standard deviations (for normally distributed data) or median and interquartile range (for nonparametric data). Normally distributed data were compared using the Student’s t-test, while nonparametric data were compared using the Mann–Whitney U test. The Pearson’s correlation coefficient (normal distribution) and the Spearman rank correlation coefficient (non-normal distribution) were used to assess the relationship between variables. A P value of less than 0.05 was considered significant.

## Results

**Table 1** shows baseline and clinical characteristics. The study demonstrated that women with dyslipidemia were older than the control group, 33 years (27-37) vs 24 years (21-26), p<0.01. No significant differences in body weight, waist circumference, hip circumference, WHR, waist-to-height ratio (WHtR), or BMI were found between both groups. However, women with dyslipidemia had a higher percentage of body fat than women with normal lipid profile (40.6% vs. 35.1%, p=0.04). Similar results were observed with BAI, where higher values of BAI (35.6%) were found in the dyslipidemia group compared to the normal lipid profile group (32.0%) (p=0.02).

**Table 1:** Baseline and clinical characteristics.

Parameter Me (Q1-Q3)	Dyslipidemia (N=14)	Normal lipid profile (N=35)	p-Value
Hypertension, n (%)	2 (14.3)	2 (5.7)	0.32
Diabetes mellitus type 2, n (%)	2 (14.3)	1 (2.9)	0.19
Hypothyroidism, n (%)	8 (57.1)	15 (42.9)	0.28
Metabolic syndrome, n (%)	8 (57.1)	6 (17.1)	0.00
Impaired fasting glucose, n (%)	2 (14.3)	5 (14.3)	0.67
Impaired glucose tolerance, n (%)	4 (28.6)	4 (11.4)	0.15
Insulin resistance, n (%)	12 (85.7)	17 (48.6)	0.02
Age (years)	33.5 (27.0-37.0)	24.0 (21.0-26.0)	p<0.01
Body weight (kg)	81.6 (75.3-101.6)	71.8 (56.7-89.)	0.19
Height (cm)	165.3 (158.5-167.5)	166.0 (161.5-169.5)	0.35
Waist circumference (cm)	100.0 (88.0-105.0)	78.5 (73.0-104.8)	0.07
Hip circumference (cm)	111.5 (105.0-117.0)	105.5 (94.0-116.0)	0.16
WHR	0.9 (0.8-0.9)	0.8 (0.8-0.9)	0.05
WHtR	0.6 (0.5-0.7)	0.5 (0.4-0.6)	0.06
Percent of body fat (%)	40.6 (37.3-44.3)	35.1 (23.7-42.5)	0.04
BMI (kg/m2)	30.4 (27.5-35.9)	25.6 (20.9-32.5)	0.07
BAI (%)	35.6 (33.4-36.9)	32.0 (26.7-35.4)	0.02

WHR, Waist-to-Hip Ratio; WHtR, Waist-to-Height Ratio; BMI, Body Mass Index; BAI, Body Adiposity Index.

Women with dyslipidemia had higher glucose levels after 60 min and 120 minutes (p=0.04 and p=0.01, respectively) in the oral glucose tolerance test. There were no significant differences in FSH, LH, SHBG, estradiol, testosterone, FAI, DHEA-S, or AMH between the dyslipidemia group and the normal lipid profile group (**Table 2**).

**Table 2:** Baseline laboratory characteristics.

Parameter	Dyslipidemia (N=14)	Normal lipid profile group (N=35)	p-Value
Fasting glucose (mmol/L)	5.4 (4.7-5.5)	4.8 (4.6-5.3)	0.09
Glucose after 60 min (mmol/L)	9.0 (8.0-10.5)	7.4 (5.7-9.2)	0.04
Glucose after 120 min (mmol/L)	7.7 (6.8-9.3)	60. (5.1-6.7)	p<0.01
Fasting insulin (pmol/L)	103.0 (94.7-172.2)	73.2 (36.2-134.9)	0.10
Insulin after 60 min (pmol/L)	696.7 (406.1-772.0)	533.3 (290.6-892.6)	0.77
HOMA-IR index	3.0 (3.1-5.2)	2.0 (1.2-4.1)	0.07
Total cholesterol (mmol/L)	6.0 (5.7-7.1)	4.3 (4.1-4.8)	p<0.01
HDL cholesterol (mmol/L)	1.4 (1.3-1.7)	1.7 (1.4-2.1)	0.14
LDL cholesterol (mmol/L)	3.8 (3.5-4.7)	2.2 (20.-2.5)	p<0.01
Non-HDL cholesterol (mmol/L)	4.5 (4.2-5.4)	2.6 (2.3-3.1)	p<0.01
Triglycerides (mmol/L)	1.7 (1.4-2.3)	0.8 (0.6-1.1)	p<0.01
TSH (mIU/ml)	2.1 (1.0-2.7)	2.1 (1.3-3.0)	0.96
CRP (mg/L)	2.0 (0.7-7.0)	1.3 (0.4-3.8)	0.18
RBC (106/uL)	4.7 (4.7-5.2)	4.7 (4.4-4.9)	0.21
WBC (103/uL)	7.3 (6.0-9.2)	6.1 (5.1-7.9)	0.14
Hb (g/dL)	14.2 (13.1-14.6)	13.8 (13.4-14.4)	0.68
HCT (%)	42.7 (40.7-43.6)	41.4 (39.8-42.8)	0.14
MCH (pg)	29.3 (28.1-29.9)	29.4 (28.4-30.4)	0.46
MCHC (g/dL)	33.5 (32.8-33.6)	33.6 (32.6-34.2)	0.36
Platelet	279.8 (237.0-347.0)	264.0 (224.0-301.0)	0.56
Arterial systolic pressure (mmHg) Arterial diastolic pressure (mmHg)	130 (120.0-140.0) 80.0 (80.0-90.0)	120.0 (110.0-130.0) 75.0 (70.0-80.0)	0.02 0.07
FSH (IU/L)	*4.38 (3.14-5.46)*	*4.15 (3.59-4.99)*	*0.95*
LH (IU/L)	*5.29 (4.65-9.28)*	*6.83 (3.71-9.51)*	*0.98*
SHBG (nmol/L)	*34.9 (26.4-55.70)*	*41.60 (24.75-61.00)*	*0.72*
Estradiol (ng/dL)	*4.85 (4.35-7.65)*	*5.30 (3.70-9.60)*	*0.94*
Testosterone (ng/dL)	*0.06 (0.05-0.09)*	*0.06 (0.05-0.07)*	*0.62*
FAI	*5.45 (3.64-7.42)*	*5.00 (2.71-8.00)*	*0.90*
DHEA-S (ug/dL)	*333.60 (267.70-410.80)*	*353.20 (272.00-409.00)*	*0.64*
AMH (pmol/L)	*45.00 (34.93-51.43)*	*48.54 (32.29-107.14)*	*0.53*

FSH, follicle-stimulating hormone; LH, luteinizing hormone; SHBG, sex hormone-binding globulin; FAI, free androgen index; DHEAS, dehydroepiandrosterone sulfate; AMH, anti-Müllerian hormone.

**Table 3** presents correlations between lipid profile parameters and anthropometric indices (BMI, BAI, and percentage of body fat). BMI and BAI did not correlate with total cholesterol levels (p=0.63; p=0.27 respectively). However, serum HDL cholesterol (respectively p<0.01; p<0.01), LDL cholesterol (respectively p=0.02 and p=0.01), non-HDL cholesterol (respectively p<0.01; p<0.01) and triglycerides (respectively p<0.01; p<0.01) correlated with BMI, BAI, and percentage of body fat.

**Table 3:** Correlation between lipid profile parameters and anthropometric indices and percentage of body fat among study group.

		R	P-value
**Total cholesterol (mmol/L)**	Body weight [kg]	0.02	0.87
	Percent of body fat [%]	0.10	0.47
	BMI [kg/m2]	0.07	0.63
	BAI [%]	0.16	0.27
	WHR	0.13	0.37
	WHtR	0.09	0.51
**HDL cholesterol (mmol/L)**	Body weight [kg]	-0.67	p<0.01
	Percent of body fat [%]	-0.66	p<0.01
	BMI [kg/m2]	-0.68	p<0.01
	BAI [%]	-0.58	p<0.01
	WHR	-0.51	p<0.01
	WHtR	-0.62	p<0.01
**LDL cholesterol (mmol/L)**	Body weight [kg]	0.31	0.03
	Percent of body fat [%]	0.36	0.01
	BMI [kg/m2]	0.34	0.02
	BAI [%]	0.37	0.01
	WHR	0.37	0.01
	WHtR	0.37	p<0.01
**Triglycerides (mmol/L)**	Body weight [kg]	0.51	p<0.01
	Percent of body fat [%]	0.56	p<0.01
	BMI [kg/m2]	0.56	p<0.01
	BAI [%]	0.51	p<0.01
	WHR	0.50	p<0.01
	WHtR	0.53	p<0.01

BMI, Body Mass Index; BAI, Body Adiposity Index; WHR, Waist-to-Hip Ratio; WHtR, Waist-to-Height Ratio.

**Figure 2:**
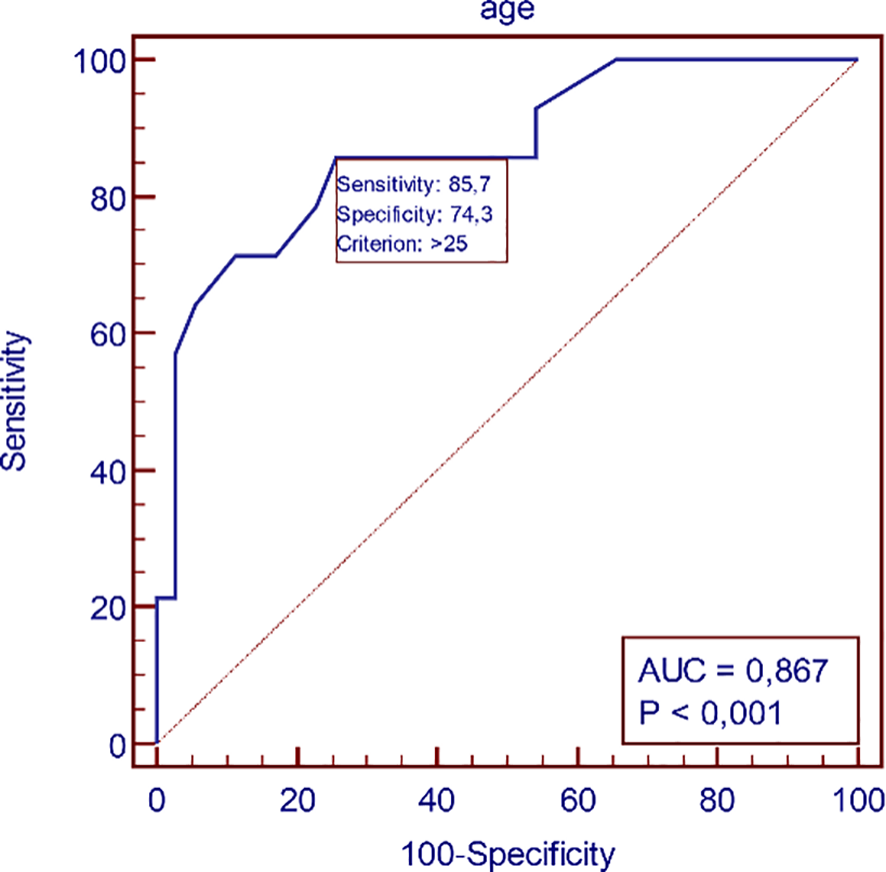
Receiver-operating characteristic (ROC) curves identifying the threshold for dyslipidemia for age.

**Figure 3:**
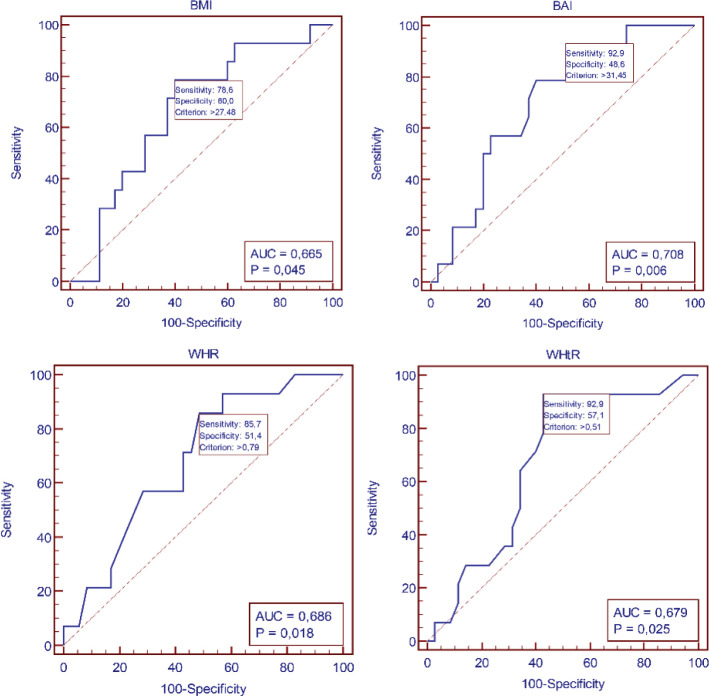
Receiver-operating characteristic (ROC) curves identifying the threshold for dyslipidemia for BMI, BAI, WHR, and WHtR.

ROC analysis demonstrated a high predictive value for age in identifying dyslipidemia. ROC analysis demonstrated poor predictive value for BMI, BAI, WHR, WHtR in identifying dyslipidemia (**Table 4**, **Figures 2**, **3**).

**Table 4:** Receiver-operating characteristic (ROC) curves identifying the threshold for existing dyslipidemia for BMI, BAI, WHR, and WHtR.

Parameters	Parameters cut-off	AUC (95%CI)	Sensitivity (%)	Specificity (%)	PPV (%)	NPV (%)	p
Age	>25	0.87 (0.74-0.95)	85.7	74.3	57	93	p<0.01
BMI	>27.5	0.67 (0.52-0.79)	78	60	44	87	0.04
BAI	>31.45	0.71 (0.56-0.83)	95.9	48.6	42	94	p<0.01
WHR	>0.79	0.69 (0.54-0.81)	85.7	51.4	41	90	0.02
WHtR	>0.51	0.68 (0.56-0.81)	92.9	57.1	46	95	0.03

AUC, area under the curve; CI, confidence interval; PPV, positive predictive value; NPV, negative predictive value; ROC, receiver-operating characteristic.

## Discussion

We aimed to assess cardiometabolic risk in women with PCOS using lipid profiles and anthropometric measures. According to the authors, despite the relatively young age of women with PCOS they are at an elevated risk for CVD. This underscores the importance of early proactive monitoring and management of CVD risk, incorporating both pharmacological and non-pharmacological strategies. However, the findings, seem to be conflicting. Interestingly, a 32-year follow-up study of the Gothenburg PCOS cohort, where participants reached a mean age of over 80 years and age-matched individuals of similar BMI, did not show an increased risk of CVD mortality or morbidity (20). These results shed new light on CVD risk in relation to confounding lipid deregulations in women with PCOS. The CVD risk of women with PCOS might be affected by factors other than the ones measured in our study. For instance, the authors of the Gothenburg study highlighted that deceased woman with PCOS had higher WHR parameters. Therefore, additional anthropometric measures might provide further insights into the CVD risk in PCOS. In contrast, a study conducted by Atanasova et al. indicated that WHtR and lipid accumulation product (LAP) are accurate indicators for predicting incident CVD in women with PCOS. In this study, the mean WHtR value in women with PCOS was significantly higher than in the control group (7). This suggests that WHtR could be a relevant anthropometric measure for assessing cardiometabolic risk in women with PCOS. Our study contributes to the existing data on metabolic risk assessment in women with PCOS. However, the discrepancies between our findings and those of other studies underscore the complexity of CVD risk factors in PCOS. The potential of additional anthropometric indices, such as WHR and WHtR, to better understand underlying mechanism of CVD risk.

Lipid metabolism constitutes one of the components of CVD risk. Additionally, the influence of glucose metabolism on CVD risk appraisal merits consideration. The authors of the study conducted an analysis of more complex anthropometric indices: Visceral Adiposity Index (VAI), Body Roundness Index (BRI), LAP, as well as atherogenic indices: Atherogenic Index of Plasma (AIP), Atherogenic Coefficient (AC), Castelli’s risk index-I, Castelli’s risk index-II, METS-IR, TG/HDL-C ratio, TyG index, TyG-BMI index, TyG-WC index in the study group. However, due to inherent inclusion of select lipid parameters within these indices, their explicit evaluation was precluded in the present study. Nonetheless, these indices demonstrated significant correlations with glucose and insulin parameters within the cohort of patients diagnosed with PCOS (21).

Among the Iranian cohort of 176 women diagnosed with PCOS and hyperandrogenism (A & B phenotypes) based on the diagnostic criteria of NIH, Rotterdam and AE-PCOS, had significantly higher WHtR and elevated TG levels compared to the healthy group (22). This highlights the association of hyperandrogenism with adverse cardiometabolic outcomes in women with PCOS.

A retrospective cohort study conducted in 8.000 young women with PCOS (mean age 28 years) revealed that these women had a 63% higher incidence of coronary artery disease (CAD) than healthy controls during 6-year of follow-up (23). Interestingly, the study found that a single factor of PCOS, such as infertility, irregular menstruation, or obesity, was not independently associated with CAD risk in this group. Instead, the authors emphasized that comorbidities such as hyperlipidemia, hypertension or diabetes might interact with PCOS in escalating CAD risk (23). This suggests that a comprehensive approach to managing cardiometabolic risk factors is essential in women with PCOS to mitigate the risk of CAD.

It has been demonstrated that higher levels of C-reactive protein (CRP) in obese women with PCOS are associated with susceptibility to dyslipidemia and diminish endothelial function in these individuals (24, 25). Considering that elevated CRP levels are known to by a major risk factor of CVD (24), it is crucial to understand its implications in women with PCOS. Lipid abnormalities among women with PCOS are commonly manifested as elevated levels of LDL-C, VLDL-C, TG, and lowered HDL-C levels. A meta-analysis of 30 studies that took into consideration BMI demonstrated that women with PCOS had higher LDL-C levels by 9.2 mg/dL, higher non-HDL-C levels by 16.3 mg/dL, and higher TG levels by 26.4 mg/dL compared to women without PCOS (26). Our study provides evidence of significant differences in lipid profiles in women with PCOS. This underscores the importance of close monitoring of lipid profiles in women with PCOS, especially in those with excess BMI or/and BAI. Furthermore, potential usefulness of BAI in predicting dysregulation of glucose metabolism has been hypothesized (27). Recognized cut-off values for BAI and BMI could serve as quick and non-expensive markers of cardiometabolic risk among women with PCOS.

Studies report heterogeneity in both CVD risk and in the clinical manifestation of PCOS associated with the PCOS diagnosis, which suggests that PCOS types appear to play a significant role in the occurrence of CVD risk (22, 28, 29).

PCOS with hyperandrogenism and ovulatory dysfunction has been proposed to pose severe risk factors for T2DM and to increase the risk of CVD in women (20, 28, 29). On the other hand, Iranian women with PCOS who had only ovulatory dysfunction and polycystic ovulatory morphology had similar cardiometabolic characteristics to healthy women (22). These findings suggest that women with PCOS who have only ovulatory dysfunction and no other features might have a lower cardiometabolic risk. The presence of hyperandrogenism and anovulation in PCOS types could lead to hyperandrogenism and hormonal imbalances, promoting IR and dyslipidemia. On the other hand, types of PCOS that are primarily defined by ovulatory dysfunction without prominent hyperandrogenism appear to have a lower risk of CVD. They are usually associated with a lower BMI and reduced abdominal obesity along with a lower prevalence of MetS and dyslipidemia (28, 29). These observations suggest that classification of PCOS may have implications for CVD risk stratification and management. Women with reproductive and hyperandrogenic PCOS clinical scenario may benefit from more vigilant monitoring and targeted interventions to address their higher cardiometabolic risk. In contrast, those with non-typical PCOS types may require a different approach, focusing on ovulatory dysfunction management and maintaining healthy lifestyle to minimize the risk.

A 17-year follow-up study of 195.675 women with PCOS who underwent deliveries compared to 71.240.633 women without PCOS reported that a diagnosis of PCOS during delivery was associated with increased cost and length of hospitalization (p<0.01). In addition, it was linked to an elevated risk of preeclampsia/eclampsia, peripartum cardiomyopathy, obesity, diabetes, and dyslipidemia (3).

We found the association of increased dyslipidemia in older women with PCOS (average 33.5 years old) compared to those with normal lipid profile (average 24 years old). This suggest that age may indeed play a role in the development of dyslipidemia in women with PCOS. As women with PCOS age, they may experience hormonal changes and metabolic changes that could contribute to an increased risk of dyslipidemia and cardiovascular complications. In addition to age, other features of PCOS have been proposed as contributing factors to diabetogenic and cardiometabolic factors. Hyperandrogenemia, low sex hormone binding globulin levels, and obesity are all known to be associated with PCOS and have been linked to an increased risk of developing diabetes and cardiovascular diseases (30). These factors may create a complex interplay that aggravates cardiometabolic risk in women with PCOS.

Recent advances in medical treatment have provided promising options for managing cardiometabolic risk in women with PCOS. Sodium glucose cotransport-2 inhibitors (SGLT-2) inhibitors and glucagon-like peptide-1 (GLP-1) receptors agonists have shown efficacy in improving diabetes management and cardiovascular outcomes (3, 31). These medications offer potential benefits for women with PCOS who may be at an increased risk of developing diabetes and CVD due to their underlying hormonal and metabolic imbalance. Overall, our study underscores the importance of early cardiometabolic risk assessment in women with PCOS, considering their increased risk to cardiovascular complications. Improving patient outcomes require targeted intervention that address the specific cardiometabolic risk factors associated with PCOS. In addition to promoting a healthy lifestyle, integrating the latest evidence-based pharmacological therapies such as SGLT-2 inhibitors and GLP-1 receptor agonist into clinical practice may improve outcomes and enhance the overall management of PCOS-related CVD. However, further research is warranted to fully elucidate the long-term benefits, safety, and potential risks of these medications in women with PCOS.

## Conclusions

Analysis of simple and rapid parameters used to assess body fat, such as BMI, BAI, WHR, and WHtR, has shown that they are poor predictors of dyslipidemia in women with PCOS. In young women with PCOS, age appears to be a more reliable predictor of dyslipidemia.

### Limitations

The conclusion drawn from this study should be interpreted in the context of its limitations. Relatively small number of participants, which may have affected the statistical power and rendered some differences statistically insignificant. However, other a substantial proportion of studies on women with PCOS are also relatively small.

Despite the limitations, our study underscores the significance of prioritizing cardiometabolic risk assessment among young women with PCOS, particularly in those with excessive body mass and abdominal visceral fat.

## Data Availability

The raw data supporting the conclusions of this article will be made available by the authors, without undue reservation.
